# Adherence to All Steps of a Pain Management Protocol in Intensive Care Patients after Cardiac Surgery Is Hard to Achieve

**DOI:** 10.1155/2017/7187232

**Published:** 2017-02-16

**Authors:** L. van Gulik, S. J. G. M. Ahlers, P. Bruins, D. Tibboel, C. A. J. Knibbe, M. van Dijk

**Affiliations:** ^1^Department of Anaesthesiology, Intensive Care and Pain Management, St. Antonius Hospital, Koekoekslaan 1, 3440 EM Nieuwegein, Netherlands; ^2^Department of Clinical Pharmacy, St. Antonius Hospital, Koekoekslaan 1, 3440 EM Nieuwegein, Netherlands; ^3^Intensive Care, Erasmus Medical Centre, Sophia Children's Hospital, Dr. Molewaterplein 60, 3015 GJ Rotterdam, Netherlands

## Abstract

*Purpose.* To investigate adherence to our pain protocol considering analgesics administration, number and timing of pain assessments, and adjustment of analgesics upon unacceptably high (NRS ≥ 4) and low (NRS ≤ 1) pain scores.* Material and Methods.* The pain protocol for patients in the intensive care unit (ICU) after cardiac surgery consisted of automated prescriptions for paracetamol and morphine, automated reminders for pain assessments, a flowchart to guide interventions upon high and low pain scores, and reassessments after unacceptable pain.* Results.* Paracetamol and morphine were prescribed in all 124 patients. Morphine infusion was stopped earlier than protocolized in 40 patients (32%). During the median stay of 47 hours [IQR 26 to 74 hours], 702/706 (99%) scheduled pain assessments and 218 extra pain scores were recorded. Unacceptably high pain scores accounted for 96/920 (10%) and low pain scores for 546/920 (59%) of all assessments. Upon unacceptable pain additional morphine was administered in 65% (62/96) and reassessment took place in 15% (14/96). Morphine was not tapered in 273 of 303 (90%) eligible cases of low pain scores.* Conclusions.* Adherence to automated prescribed analgesics and pain assessments was good. Adherence to nonscheduled, flowchart-guided interventions was poor. Improving adherence may refine pain management and reduce side effects.

## 1. Introduction

International clinical guidelines recommend systematic evaluation of pain in patients in intensive care units (ICUs) [1]. Pain education and pain management protocols helped reduce pain scores in patients in mixed ICUs [2] and in postcardiac surgery patients [3, 4]. However, adherence to guidelines in general is often poor [5, 6] and the same holds true for pain management in the ICU specifically [7, 8]. In a prospective, observational study in 44 ICUs in France pain assessment was performed in 42% of patients, while 90% of patients received opioids [7]. In a study when caregivers knew that their pain assessments were monitored, still less than 50% of ICU patients were adequately assessed [8].

We previously implemented a pain management program for postcardiac surgery patients in the ICU, thereby reducing the occurrence of unacceptable pain (NRS ≥ 4) from 41% to 23% [4]. However, still 46% of patients experienced at least one event of unacceptable pain during ICU stay. In order to improve further pain management, we then studied two different dosages of morphine for the prevention and treatment of procedural pain in patients after cardiac surgery in a randomized controlled trial [9]. In addition, we evaluated in that study adherence to the pain protocol used to assess and treat pain, in order to investigate if pain management in rest could be ameliorated.

The aim of the current study was to determine the adherence to the unit's postoperative pain management protocol in adult patients in the ICU after cardiac surgery in terms of the administration of analgesics, the number and timing of pain assessments, and adjustment of analgesics upon unacceptably high (NRS ≥ 4) and low (NRS ≤ 1) pain scores.

## 2. Material and Methods

### 2.1. Patients and Study

In this analysis, data were retrieved from a study on postoperative pain management (ClinicalTrials.gov identifier NCT00558090) that took place in a 30-bed mixed ICU from February 2008 until November 2008. In that study both pain levels in rest and pain levels upon an unavoidable medical procedure were measured. Procedural pain levels in the patients upon two randomized dosages of morphine are described elsewhere [9]. The study protocol was approved by the local Ethics Committee of the St. Antonius Hospital, a large teaching hospital in Nieuwegein, Netherlands (approval number R0715A, 7 November 2007). Written informed consent was obtained from 128 patients before elective cardiac surgery. Three patients were excluded because they were not admitted to this ICU postoperatively and one patient died within hours after the admission to the ICU. Thus the study group numbered 124 patients.

### 2.2. Pain Protocol for Treatment of Postoperative Pain

**Figure 1:**
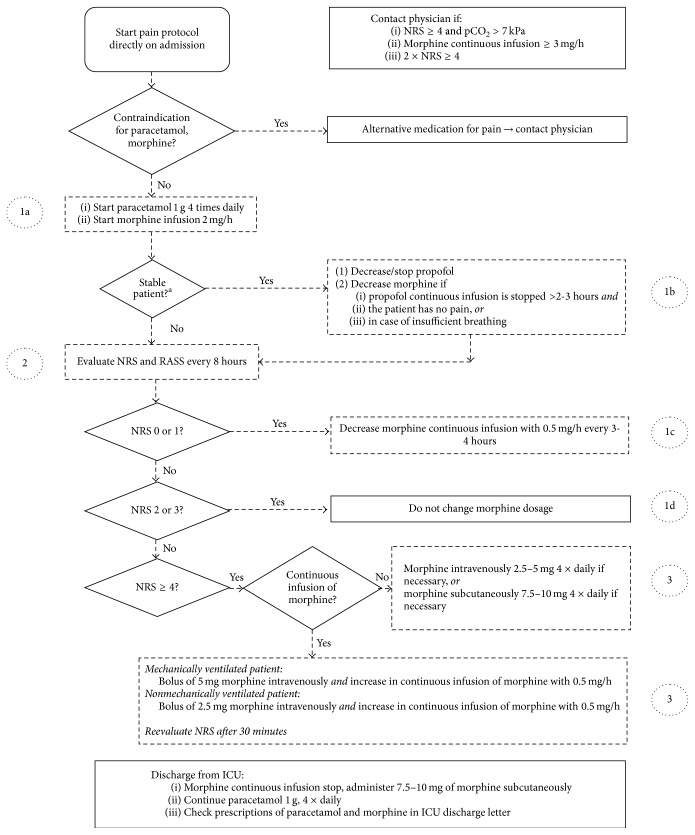
Pain management protocol after cardiac surgery. ^a^Stable patient: haemodynamically stable, acceptable leakage through thoracic drains, adequate time after muscle relaxation, and adequate core temperature; NRS: numeric rating scale, RASS: Richmond Agitation and Sedation Scale, ICU: intensive care unit, and 1(a, b, c, d), 2, and 3: items concerning adherence to the pain protocol referred to in the Results.

In our ICU, a pain protocol consisting of paracetamol 4 grams daily and a continuous intravenous infusion of morphine for all patients after cardiac surgery had been in place for one year before start of the study (Figure 1). The dosing of paracetamol and morphine infusion could be reduced by the anesthesiologist if this was deemed appropriate. Twice a year and before start of the study, physicians and nurses had been (re)trained in assessing pain and in providing adequate analgesia [4]. Pain was measured with the 11-point numeric rating scale (NRS) in which “0” represents no pain and “10” represents the worst pain imaginable [10, 11] and which has been proven to be valid in ICU patients [12]. Scores 0 and 1 indicate low, scores 2 and 3 indicate acceptable, and scores of 4 or more indicate unacceptable pain [13]. Pain scores were preferably reported by patients themselves. The NRS had been explained to all patients the day before surgery. Intubated patients could either nod when the correct pain score was said out loud by the nurses (nurses counted up from 0 to 10) or point out on the visually enlarged VAS (Visual Analogue Scale) so that these patients could also score their pain intensity. When a patient was not able to report pain, for example, due to sedation, the attending nurse applied the NRS. Nurses based the NRS pain scores on behavioral signs of pain such as used in the Behavioral Pain Scale [12]. Nurses received automated reminders to ask patients to provide a pain score at least three times a day: at 8:00, at 16:00, and at 0:00 [4]. Additional pain scores were to be provided within half an hour after the recording of unacceptable pain scores (NRS ≥ 4) (Figure 1). Extra pain scores could be recorded at any time on nurses' own initiative.

### 2.3. Data Collection

Patient characteristics, type of surgery and data on length of stay in the ICU, and duration of mechanical ventilation were prospectively registered. Pain scores and administered medication during the first 72 hours in the ICU were retrieved from the patient data monitoring system (PDMS). The 72-hour time frame was chosen as most patients are discharged from the ICU within this period.

Omissions to administer prescribed medication, to assess scheduled pain scores, to reassess and intervene pharmacologically upon NRS ≥ 4, to taper or stop continuous infusion of morphine earlier than the protocol dictated, and to not taper continuous infusion of morphine upon NRS ≤ 1 after at least 2-3 hours without sedation were registered. In case of deviations from the protocol, electronic medical files of the patients were searched for reasons for these deviations.

### 2.4. Data Analysis

Descriptive analyses were performed using IBM SPSS Statistics (version 19.0 for Windows; SPSS, Chicago, IL, USA). Patient characteristic and clinical variables are expressed as frequencies with percentages (%) or median with interquartile range (IQR) where appropriate. Chi square tests were used to test categorical data.

## 3. Results

### 3.1. Patients and Data

Demographic characteristics, type of surgery, and duration of mechanical ventilation are shown in Table 1. Median age was 69 years and males predominated (92/124, 74%). Median length of stay in the ICU after cardiac surgery was 47 hours [IQR 26 to 74 hours].

### 3.2. Duration and Dosage of Analgesic Treatment (Figure 1, Numbers 1(a, b, c, d))

All patients received paracetamol. Paracetamol was prescribed and administered 4 grams daily, according to protocol, in 109/124 (88%) patients. 15/124 (12%) patients received paracetamol at a lower dosage (range 2.5 to 4 grams). All patients received a continuous infusion of morphine directly after ICU admittance (Figure 1, 1a). This morphine infusion was stopped earlier than protocolized as described under 1b, 1c, and 1d in Figure 1 in 40 (32%) patients.

### 3.3. Pain Scores

In 124 patients, 920 NRS scores were recorded with a median of 7 [IQR 5 to 10] measurements per patient. Patients were able to provide self-reported pain scores in 570/920 (62%) of the scores. Scheduled pain scores were performed 702 times of the maximum scheduled 706 scores, which equals an adherence of 99%. Extra pain scores, for example, on suspicion of pain and reassessment after cessation of sedation, accounted for 24% of all recorded pain scores (218/920).

**Figure 2:**
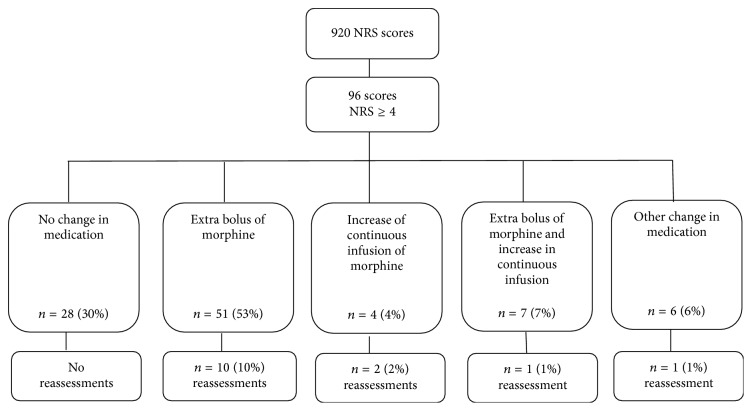
Actions upon pain scores with NRS ≥ 4. NRS: numeric rating scale.

Ten per cent (96/920) of NRS scores were ≥4, signifying unacceptable pain (Figure 2) and 59% (546/920) of NRS scores were ≤1, signifying low/no pain. Patients whose morphine was stopped earlier than protocolized had significantly more often unacceptable pain scores afterwards than patients with morphine treatment according to protocol, that is, 71% versus 33%, respectively (*p* = 0.002).

### 3.4. Actions upon Unacceptably High (NRS ≥ 4) and Low (NRS ≤ 1) Pain Scores

In 28 of the 96 cases of NRS scores ≥ 4 (29%), medication was not changed whereas protocol dictated this (Figure 2). In 51 cases (53%), an extra bolus of morphine was administered, in 4 cases the continuous infusion of morphine was increased, and in 6 cases another change in medication was made (extra paracetamol *n* = 1, decrease of continuous morphine infusion *n* = 3, or a bolus of morphine combined with a decrease of continuous morphine *n* = 2). Seven patients each received once the protocolized combination of an extra bolus of morphine with an increase of continuous morphine (Figure 2). Overall, in 14/96 cases (15%) NRS reassessment after NRS ≥ 4 was performed as dictated by protocol; all of these NRS were ≥4.

**Figure 3:**
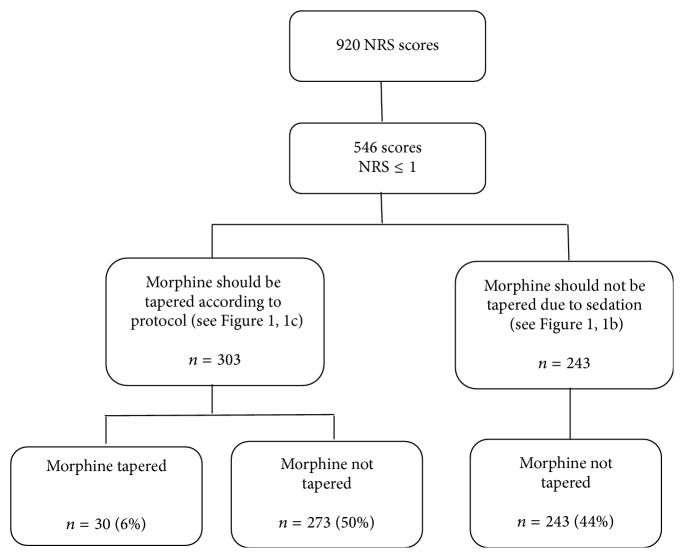
Actions upon pain scores with NRS ≤ 1. NRS: numeric rating scale.

In 273 of the 303 cases (90%) where morphine should have been tapered, because of NRS scores ≤ 1 and discontinuation of propofol (see 1b-1c in Figure 1), morphine was continued. In 243 of the 243 cases (100%) of NRS ≤ 1 where morphine should not have been tapered, that is, within 2-3 hours after discontinuation of propofol, morphine was continued according to protocol. As such, in a total of 50% (30 + 243)/546 of the cases of NRS scores ≤ 1 the protocol was followed (Figure 3).

### 3.5. Reasons for Deviation from the Pain Protocol

Reasons for the lower paracetamol dosage than recommended, prescribed to 14/124 patients (11%), were not documented except for one patient who missed one dose of paracetamol because he was in the operating room for a resternotomy at the scheduled time of administration.

In the nurses' notes motivations for terminating or tapering continuous morphine earlier than by protocol or for not administering extra morphine upon an unacceptable pain score could be found in 43 times (Table 2). In 7 of 273 (3%) cases where morphine should have been tapered upon a NRS of 0 or 1, the nurse had documented why this was not done (Table 2).

## 4. Discussion

In this study we evaluated the level of adherence to the pain protocol for patients in ICU patients after cardiac surgery. Adherence to scheduled pain assessments was excellent, as 99% was performed. Adherence to initiation of prescribed analgesics with automated reminders via the PDMS at administration times was also good. However, adherence to the nonscheduled items of the protocol that rely on the initiative of the caregiver was less adequate. Unacceptable pain was followed by reassessment of pain in 15% and administration of additional morphine in 65% of the events. Furthermore, in only 10% of low pain scores, morphine was tapered. Time to discharge to the ward was not delayed due to respiratory depression, nor were there any serious adverse events such as need for reintubation in the studied patients. We may therefore conclude that the use of this pain protocol was safe in our ICU. Although overall pain management was good with 90% of pain scores reflecting acceptable pain intensity, there may be room for improvement as adherence to different items of the pain protocol varied from excellent to poor.

Hospital departments in general, and intensive care units in particular, are still struggling with nonadherence to guidelines in general [5, 6] and more specifically to pain management [7, 14]. Diby et al. [3] found that only 70% of scheduled pain assessments in the ICU after cardiac surgery were carried out. Pain was assessed in only 40% of patients in an observational study in 44 ICUs in France, although 90% of patients received opioids [7]. A study on nurses' knowledge and management of pain after cardiac surgery patients reported moderate to severe pain, even though only 47% of the prescribed dose of analgesics was given [15]. These studies did not use automated reminders to assess pain and to administer analgesics in contrast with our pain protocol, resulting in excellent adherence to scheduled pain assessments and administration of prescribed analgesics in our ICU.

However, only 15% of unacceptable high pain scores were followed by reassessments of pain. Nonadherence to obliged reassessments was previously reported by others as well [3, 14, 16]. Reassessments after recorded pain events were performed in 36% in a pediatric ICU [14], in 45% in ICU patients after cardiac surgery [3] and in 60% of recorded pain events in critically patients [16]. Bucknall et al. suggested 3 explanations for nurses not performing reassessments in postoperative patients: their busyness or workload, the lack of knowledge concerning the importance of adequate pain management and pharmacologic properties of analgesics, and, thirdly, patients not reporting their pain [17]. In our study, both nurses and patients were informed on the importance of adequate pain management due to the fact that they were participating in a clinical trial. We did not investigate the nurses' workload. Another explanation could be that nurses did not feel the need to reassess because they treated the pain through a pharmacological intervention.

Adherence to pharmacological interventions in our study upon high or low pain scores, both nonscheduled parts of the pain protocol, was poor as well. Part of these deviations from the protocol were justifiable as reported explanations were mostly related to side effects of morphine. However, explanations for all deviations from the protocol should be recorded in order to uncover pitfalls in the protocol. As patients in whom continuous morphine was stopped earlier than protocolized experienced unacceptable pain significantly more often than patients in whom protocol was not violated (71% versus 33% resp., *p* = 0.002), the protocol should be adjusted to suggest alternative analgesics and antiemetic medication for patients suffering from side effects.

In order to prevent side effects of morphine such as respiratory depression, nausea, and vomiting, the protocol dictated that continuous infusion of morphine had to be tapered in patients with no or low pain scores (NRS ≤ 1). This was executed in 10% of required cases. This lack of titrating medication to effect was seen even more extremely in the aforementioned study in 44 ICUs in France [7] where fentanyl and morphine dosages were not changed during the week. A reason for not tapering continuous morphine at low pain scores could be that nurses took other issues into account than a pain score at a scheduled moment, such as pain while coughing or moving or an upcoming potentially painful procedure. Furthermore, nurses could be reluctant to taper morphine because they were concerned this would alter the comfortable state of the patient. Similar findings are reported for sedatives. In a study by Dodek et al. nurses were more likely to increase sedatives than decrease them [18]. The reason had to do with fear of agitation when sedation was lowered too much and depended on physician availability and other organizational features. In order to improve adherence to pain management guidelines, Ista et al. recommended interactive education sessions, involving local champions and giving feedback on individual or unit level to increase the intrinsic motivation of professionals. Finally, in the current study, the patient data monitoring system did not remind nurses that morphine may be tapered when low pain scores were recorded.

A computerized version of a guideline was reported to significantly improve timeliness of measurements compared with a paper-based version in glucose level regulation for critically ill patients [19]. Another study showed that an electronic visual feedback tool to monitor adherence to quality indicators in intensive care medicine significantly increased adherence rates for pain and delirium monitoring and implementation of the weaning protocol [20], even though feedback on performed pain and delirium monitoring was not immediately available to medical staff, but with a delay of 24 hours. Feedback upon recorded pain scores to adjust analgesia via automated reminders, such as a red flag for extra pain assessment shortly after an unacceptable high pain score or a request to record the reason for protocol violation, may improve protocol adherence.

Some limitations of this study should be addressed. The fact that patients participated in a clinical trial evaluating pain may have induced a bias; adherence to the pain protocol may have been augmented due to extra reminders of the protocol both by the PDMS and by the researchers as part of the clinical trial. The current pain protocol did not include pain management for procedural pain other than an extra morphine dose before the removal of thoracic drains and/or turning on the morning after surgery [9]. On the other hand, although these procedures are known to be potentially painful, in many ICUs, analgesia for procedural pain is still not part of standard care [7]. Fortunately, pain protocols for procedural pain are recommended and effective to reduce pain and adverse events [21]. The current study did not research an unspoken resistance to the concept of the protocol as explanation for nonadherence to the pain protocol. Franck and Bruce suggested that lack of evidence based improvement in outcome may be an underlying reason for resistance to guideline adherence in pediatric pain management [22]. In ICU patients however improvement in clinical outcome, for example, duration of mechanical ventilation [2, 23, 24], nosocomial infections [2], and ICU length of stay [23, 24], has been demonstrated after the implementation of pain management protocols and should therefore not be a reason for resistance to a pain protocol.

## 5. Conclusions

We conclude that adherence to scheduled pain management was good and overall pain management adequate. Unfortunately, adjustment of continuous infusion of morphine upon pain scores (high and low) and reassessment of pain after unacceptably high pain scores was poor. Better adherence to these items, such as reassessments and adjustment of morphine upon low and high pain scores, may further improve pain management and reduce side effects.

## Figures and Tables

**Table 1:** Patient characteristics.

	*N* = 124
Male, *n* (%)	92 (74%)
Age, years, median [IQR]	69 [64 to 78]
BMI, kg/m^2^, median [IQR]	27 [24 to 29]
Type of surgery, *n* (%)	
CABG and valve surgery	55 (44%)
Aortic surgery	25 (20%)
CABG	19 (15%)
Valve surgery	25 (20%)
LOS ICU, hours, median [IQR]	47 [26 to 74]
Duration of mechanical ventilation, hours [IQR]	10 [7 to 15]

IQR = interquartile range, BMI = body mass index, CABG = coronary artery bypass graft, LOS = length of stay, and ICU = intensive care unit.

**Table 2:** Reasons for deviating from the protocol.

Reasons for terminating or tapering morphine earlier than protocolized (*N* = 43)	*n*

Respiratory depression^*∗*^	14
Sleepiness	11
Too slow awakening after cessation of sedation	9
Nausea	2
Discrepancy between patients' high pain score and behavior according to the nurse	2
Hypotension	1
Delirium suspected to be caused by morphine	1
Refusal of a patient to receive more morphine	1
Decrease of pain immediately after removal of chest tubes	1
Planned extubation directly after pain scoring	1

Reasons for not tapering morphine infusion upon NRS 0 or 1 (*N* = 7)	*n*

Painful in the previous shift	2
Pain assessments were only within a few hours after surgery	2
Hypertension	2
Low pain scores in rest, but still painful while moving	1

^*∗*^Respiratory depression was defined as respiratory rate of less than 10/min or pCO_2_ of 7 kPa or more. None of the patients with respiratory depression was reintubated or required naloxone.
